# Supplementary material for: Molecular Insights into the Role of Sterols in Microtuber Development of Potato Solanum tuberosum L
Source: Plants (Basel). 2024 Aug 27;13(17):2391. doi: 10.3390/plants13172391 (PMC11397162; doi:10.3390/plants13172391)
Supplement: Supplementary file 1 [file plants-13-02391-s001.zip › plants-3138774-supplementary.pdf]

Supplementary Material

Table S1. Number of interactions and combined score values calculated for interactions between up-regulated genes of sterols biosynthesis pathway.

	Number of interactions	FACKEL	SMO2	CPI1	CYP51	SMO1	SMT1	3BETA HSD/ D2	StSSR2	MVD2	HMG3	BAS	CAS1	STE1	SMT2	FPS1	SI1
FACKEL	14	NI	0.792	0.830	0.995	0.792	0.770	0.768	0.942	0.679	0.460	0.434	0.807	0.914	0.400	0.611	0.984
SMO2	12	0.792	NI	0.416	0.972	NI	0.597	0.979	0.498	0.432	NI	0.707	0.781	0.945	0.949	NI	0.946
CPI1	10	NI	0.416	NI	0.969	0.417	0.738	0.506	NI	0.635	NI	NI	0.817	0.595	0.435	0.577	0.837
CYP51	9	NI	0.972	NI	NI	NI	NI	NI	0.960	0.663	0.504	0.948	0.962	0.938	0.759	0.430	0.580
SMO1	8	NI	NI	NI	0.972	NI	0.511	NI	0.498	0.432	NI	0.707	0.710	0.924	NI	NI	0.489
SMT1	7	NI	0.597	NI	0.804	0.955	NI	0.454	0.934	NI	NI	NI	0.965	0.623	NI	NI	0.953
3BETAHSD/D2	7	NI	0.979	NI	0.922	0.979	NI	NI	NI	NI	NI	0.643	0.643	0.802	0.454	NI	0.400
StSSR2	7	0.942	0.498	NI	0.960	0.498	0.934	NI	NI	NI	5	0.432	0.447	NI	NI	NI	0.954
MVD2	5	NI	0.432	NI	NI	NI	NI	NI	NI	NI	0.761	NI	0.808	0.610	NI	0.955	0.437
HMG3	4	0.460	NI	NI	0.504	NI	NI	NI	NI	0.761	NI	NI	NI	NI	NI	0.612	NI
BAS	4	NI	0.707	NI	NI	NI	NI	NI	0.432	0.443		NI	NI	0.692	NI	0.708	NI
CAS1	3	NI	0.781	NI	NI	NI	NI	NI	0.447	NI	NI	NI	NI	NI	NI	0.794	0.776
STE1	2	NI	0.945	NI	NI	NI	NI	NI	NI	NI	NI	NI	0.842	NI	NI	NI	0.961
SMT2	2	NI	0.949	NI	NI	NI	NI	NI	NI	NI	NI	NI	NI	0.536	NI	NI	0.929
FPS1	2	NI	NI	NI	NI	NI	NI	NI	NI	NI	0.612	NI	NI	NI	NI	NI	0.711
SI1	1	NI	0.946	NI	NI	NI	NI	NI	0.954	NI	NI	NI	NI	NI	NI	NI	NI
